# Substituting sitting with standing and walking in free-living conditions improves daily glucose concentrations in South Asian adults living with overweight/obesity

**DOI:** 10.1007/s00421-025-05919-7

**Published:** 2025-08-05

**Authors:** Kamalesh C. Dey, Julia K. Zakrzewski-Fruer, Lindsey R. Smith, Rebecca L. Jones, Benjamin D. Maylor, Thomas E. Yates, Daniel P. Bailey

**Affiliations:** 1 Institute for Sport and Physical Activity Research, School of Sport Science and Physical Activity, University of Bedfordshire, Bedford, UK; 2 Centre for Preventive Neurology, Wolfson Institute of Population Health, Barts and the London School of Medicine and Dentistry, Queen Mary University of London, London, UK; 3 Institute of Education, Arts and Community, Federation University, Victoria, Australia; 4 Sport, Physical Activity, and Human Performance Research Group, School of Psychology, Sport Science and Wellbeing, University of Lincoln, Lincoln, UK; 5 Department of Sport, Health and Exercise Sciences, Department of Life Sciences, Brunel University of London, Uxbridge, UB8 3PH UK; 6 Centre for Physical Activity in Health and Disease, College of Health, Medicine and Life Sciences, Brunel University of London, Uxbridge, UK; 7 Diabetes Research Centre, Leicester General Hospital, University of Leicester, Leicester, UK; 8 Diabetes Research Centre, University of Leicester, Leicester, LE5 4PW UK; 9 NIHR Leicester Biomedical Research Centre, University Hospitals of Leicester NHS Trust, Leicester, LE1 5WW UK

**Keywords:** Cardiometabolic health, Flash glucose monitoring, Prolonged sitting, Physical activity, Obesity

## Abstract

**Background:**

Controlled laboratory studies have demonstrated that breaking up sitting can reduce postprandial glucose in South Asian adults. This study examined the effects of substituting sitting with standing and walking on interstitial glucose in South Asian individuals under free-living conditions.

**Methods:**

South Asian adults (*n* = 14 [50% male]; body mass index 26.5 ± 0.8 kg·m^−2^) aged 41 ± 3 years completed two, 4-day regimens in a counter-balanced order: (1) SIT (restrict walking and standing to ≤ 1 h/day each) and (2) SITless (substitute ≥ 5 h/day of sitting with ≥ 3 h of standing and ≥ 2 h of walking, and interrupt sitting every 30 min). Interstitial glucose was measured using Flash glucose monitoring. Sitting and physical activity were measured with the activPAL3. Outcomes were compared between regimens using linear mixed models.

**Results:**

Interstitial glucose net incremental area under the curve (iAUC) for waking hours was lower by − 9.2 mmol L^−1^·16 h^−1^ (95% Confidence Interval [CI]: − 18.1, − 0.3) in SITless than SIT (*p* = 0.04), while lunch postprandial glucose iAUC was significantly lower by -1.0 mmol L^−1.^2 h^−1^ (95% CI − 1.8, 0.2) in SITless (*p* = 0.02). There were no significant differences in other 24 h or 16 h glucose metrics (*p* ≥ 0.06). Compared to SIT, sitting was lower by − 3.6 h/day (95% CI − 4.9, − 2.3) in SITless (*p* < 0.01). Standing and stepping time were higher by 1.9 h/day (95% CI 0.6, 3.2) and 1.6 h/day (95% CI 1.2, 2.1) in SITless (*p* ≤ 0.01).

**Conclusions:**

Substituting sitting with standing and walking under free-living conditions can be used to effectively attenuate glycaemia during waking hours, but not across 24 h, in South Asian adults.

**Clinical trial registration:**

NCT04645875..

**Supplementary Information:**

The online version contains supplementary material available at 10.1007/s00421-025-05919-7.

## Introduction

South Asian individuals are considered an indigenous population originating from the Indian subcontinent, including Bangladesh, India, Pakistan, Sri-Lanka, and Nepal (Jalal et al. 2019). This ethnic group is at increased risk of cardiovascular disease (CVD) and Type 2 diabetes compared with Caucasians (Hanif and Susarla 2018; Misra and Khurana 2011), which could be in part due to excess internal body fat (Bays et al. 2022a). Obesity is a primary contributor to impaired cardiometabolic health in South Asian individuals (Misra et al. 2019). Therefore, South Asian individuals with overweight and obesity require targeted interventions to improve cardiometabolic health.

Populations living in developed countries spend 55–81% of their waking time in device-measured sedentary behaviour (Ekelund et al. 2020; Matthews et al. 2023). Higher sedentary time is associated with an increased risk of CVD, Type 2 diabetes, and all-cause mortality in the general population (Bailey et al. 2019; Wu et al. 2023). South Asian individuals spend a large proportion of their waking day sedentary, particularly when measured using device-based methods (~ 9 h/day) (Dey et al. 2021). This may increase their risk of cardiometabolic disease, especially in those with overweight or obesity (Ahmad et al. 2017).

Prolonged bouts of sitting lead to acute elevations in postprandial glucose, which may be due to the lack of muscular contraction-mediated glucose uptake and impaired insulin action (Dempsey et al. 2018; Homer et al. 2019). Interrupting sitting with regular bouts of standing, walking and simple resistance exercises can attenuate cardiometabolic risk markers under controlled laboratory conditions in Caucasian participants with overweight, obesity and Type 2 diabetes (Dempsey et al. 2016; Dunstan et al. 2012; Henson et al. 2016; Hawari et al. 2016). In the limited literature investigating the effects of interrupting sitting in South Asian participants, 5-min bouts of light-intensity walking every 30-min attenuated postprandial glucose, triglycerides (TAG), insulin, and metabolic load index (MLI) (Dey et al. 2024; Yates et al. 2020). Yet, the samples were described as having mixed weight status, which may be expected to increase variability in responses (Henson et al. 2020) and limit application to South Asians with overweight and obesity. Furthermore, free-living situations are distinct from controlled laboratory-based interventions and require independent consideration as they provide greater ecological validity and practical application. This is especially important as sitting may not reduce to the same extent in less controlled free-living situations.

With technological advancements in continuous glucose monitoring (CGM), including Flash Glucose Monitors (FGM) that do not require user calibration, there is opportunity to assess 24-h glucose profiles with greater accuracy under free-living conditions (Rahmi et al. 2021). In free-living studies that have used CGM, improvements in continuous glucose concentrations have been reported in response to substituting sitting with standing and light-intensity physical activity (LPA) in Caucasian participants (Duvivier et al. 2017a; Smith et al. 2021), whereas others demonstrated no effects (Bailey et al. 2022; Blankenship et al. 2019). The effects of substituting sitting with standing and physical activity in South Asian individuals under free-living situations warrant investigation to inform the relevance of this behavioural approach for improving cardiometabolic health. The current study aimed to investigate the effects of substituting sitting under free-living conditions in South Asian adults with overweight and obesity on continuous glucose profiles. It was hypothesised that substituting sitting with standing and walking would attenuate daily glucose concentrations.

## Methods

### Study overview

This was a randomised crossover design study with two activity regimens (SIT and SITless) undertaken in free-living conditions. The study was conducted according to the Declaration of Helsinki principles and approved by the University of Bedfordshire Institute for Sport and Physical Activity Research Ethics Committee (2020ISPAR006). Participants provided written informed consent to take part. The study was conducted and reported in line with the Consolidated Standards of Reporting Trials guidance (Moher et al. 2010). The trial was registered with clinicaltrials.gov/(NCT04645875). Experimental regimen order was randomised using an online computer-generated randomisation method (https://www.randomizer.org/). Following a preliminary testing visit and successful try-out-day, participants completed two experimental regimens, each lasting 4 days, with a 3 day washout period between to minimise potential carryover effects.

### Participants

South Asian adults living in the UK aged 18 to 75 years living with overweight or obesity (BMI > 23 kg m^−2^) were eligible to take part. The BMI thresholds were aligned with overweight/obesity-related comorbidities among South Asian populations (Bays et al. 2022b; Misra 2015) and calculated as body mass (kg)/height (m)^2^. ‘South Asian’ was defined as anyone identifying themselves as South Asian or British South Asian (Bays et al. 2022a). Exclusion criteria were self-reported CVD, current diagnosis of COVID-19, diabetes, a known blood-borne disease, pregnancy, recent or current smoker and other health issues (e.g., neurological disorders) or physical limitations that might limit the ability to perform the required volume of LPA. Participants were recruited from the local community (Luton and Bedford, UK) using adverts through social media posts (e.g., Facebook and Twitter) and distribution of flyers.

#### Sample size calculations

*A-priori* sample size was calculated using G*Power version 3.0.10 (Faul et al. 2007). The primary outcome was postprandial glucose (mmol L^−1^) with the mean effect size taken from previous lab-based studies in participants with overweight and obesity (*d* = 0.9; Dey et al. 2024; Dunstan et al. 2012; Henson et al. 2016). A total of 11 South Asian individuals living with overweight/obesity were required to achieve 80% power with an alpha value of 5%. Recruitment targeted 14 participants to allow for 20% dropout.

### Preliminary testing

Due to COVID-19 pandemic restrictions, anthropometric measurement tools, including a stadiometer (Harpenden 98.602, Holtain Ltd., Crymych, UK), a measurement tape, digital weighing scales (Tanita, BWB0800, Allied Weighing, UK), activity monitor (activPAL3 micro; PAL Technologies, Glasgow, UK), and a Freestlye Libre FGM with sensor (Abbott Laboratories, Maidenhead, England) were delivered to the participant’s home or workplace. Participants were then guided through taking their own measurements during a video call following standardised methods. Participants were advised to remove their shoes and heavy outer clothing before measuring their height and weight. Height (cm) was measured to the nearest 0.1 cm using a stadiometer. Body mass (kg) was measured to the nearest 0.1 kg. Waist circumference (cm) was measured at minimal inspiration to the nearest 0.1 cm (Lohman et al. 1988). Instructions and demonstration of activities (e.g., walking at a slow pace, walking on the spot, jumping up and down, and standing) for the experimental protocol were provided, as described below.

#### Try-out day

Participants performed a try-out day of the SITless regimen after preliminary testing and before the first experimental regimen. Participants were only eligible for inclusion if they were able to adhere to the experimental protocol (i.e., substitute a minimum of 5-h/day sitting with ≥ 2 h of self-perceived light walking and ≥ 3 h of standing), assessed via monitoring with an activPAL. Participants were provided with a second attempt if required.

### Experimental protocol

**Fig. 1:**
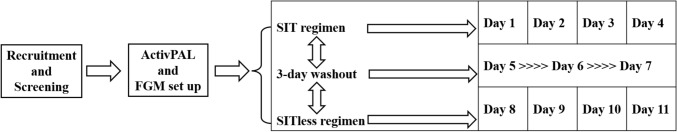
Schematic diagram of the experimental protocol

After the try-out day, participants were set up with the FGM and activPAL, which were both worn throughout the experimental period. Setup was completed 24 h before the commencement of the first experimental regimen and subsequently worn for 11 days (i.e., 2* 4 day regimens and 3 days washout). Regimen 1 (SIT or SITless) took place over 4 days (day 1–4: Monday to Thursday), followed by a 3-day washout period (day 5–7: Friday to Sunday), followed by regimen 2 (SIT or SITless) for 4 days (day 8–11: Monday to Thursday); see Fig. 1. Participants were informed of the order of their experimental regimens and provided verbal and written guidance on how to complete the protocols. During each regimen, participants were provided with a stopwatch (Quantum, 5501, Cranlea, UK) and a pedometer (Omron Walking Style IV Pedometer BLK, HJ-325-EW, Omron, Kyoto, Japan) and asked to keep a record of non-sitting time and steps in an activity logbook to encourage adherence to the protocols. The experimental regimens were as follows:

#### SIT regimen

Participants were instructed to restrict walking to ≤ 1 h/day and standing to ≤ 1 h/day during their waking day. Participants were allowed to perform their daily activities, including cooking and other household activities, within the 2-h of permitted walking and standing.

#### SITless regimen

Participants were instructed to substitute a minimum of 5 h/day sitting with ≥ 3 h of standing and ≥ 2 h of self-perceived LPA in addition to interrupting their sitting for 2–5 min every 30 min. A list of activities (including walking at a slow pace, walking on the spot, jumping up and down, and standing) was provided as examples of activities that participants could perform during this regimen. Participants received feedback on their non-sitting time and the number of light and moderate-intensity steps from the try-out day. They were guided towards achieving compliance in this regimen by identifying potential activities they could engage in to interrupt sitting, reviewing their number of sitting interruptions and aiming to accumulate approximately 12,000 steps per day (6000 steps being equivalent to approximately 1 h moderate-intensity walking) (Marshall et al. 2009).

### Control of diet and physical activity

Participants were asked to refrain from food and drink containing alcohol for 24 h before and to avoid performing structured exercise for 48 h before the experimental protocol commenced and throughout the entire experimental period (a total of 11 days). A kitchen scale (Salter Disc Electronic Kitchen Scale, Ho-Medics Group Ltd, UK) was provided so participants could weigh and record all food and beverage consumption in a diary during the first 4-day regimen and replicate this dietary intake exactly during the second 4-day regimen (Bailey et al. 2022; Duvivier et al. 2017b). Participants were instructed to consume a standardised diet (at least 50% carbohydrate) in order to reduce dietary heterogeneity within the study population. Participants were advised that the meals be spaced evenly throughout the day with 3 to 4 h intervals, with snacks consumed between meals, to ensure three distinct and consistent glucose responses across each day.

### Outcome measures

#### Flash glucose monitoring

The FGM was used to measure interstitial glucose concentrations on a continuous basis (1 min intervals stored every 15 min) throughout the experimental regimen. After the successful try-out day, an FGM sensor was inserted subcutaneously at the midline of the back of the upper arm following manufacturer guidelines. The FGM provides valid and accurate measurements of interstitial glucose for up to 14 days that correspond with capillary plasma glucose, regardless of participants’ characteristics (e.g., age, ethnicity, and weight status) (Elidottir et al. 2021; Sekido et al. 2017). Interstitial glucose concentrations (mmol L^−1^) were measured from the subcutaneous tissue’s interstitial fluid every minute. The data was averaged for each 15-min period and stored within an FGM reader before being exported into Microsoft Excel at the end of the experimental protocol.

FGM data was processed using a custom R script within RStudio software (version 1.4.1103, Boston, USA). During the activity regimen, days in which the FGM receiver recorded data > 70% of the 24 h period were classified as valid (Battelino et al. 2019). After an automated estimate of valid FGM days, RStudio summary output variables were produced for 24 h and waking hours glucose profiles in Microsoft Excel based on metrics recommended for summarising CGM data (Battelino et al. 2019). Waking hours were identified individually for each participant from activPAL data and then normalised to a 16 h day. These metrics were: (1) waking glucose concentration (mmol L^−1^), (2) mean glucose concentrations (mmol L^−1^), (3) glycaemic variability (i.e., glucose coefficient of variation [CV%]), (4) time in range (3.9–10.0 mmol.L^−1^), (5) time above range (> 10.0 mmol.L^−1^[hyperglycaemia] %), (6) time below range (< 3.0 mmol.L^−1^ [hypoglycaemia] %), (7) net incremental area under the curve (iAUC), calculated by subtracting waking glucose concentration for each day from total AUC. Each of these variables was calculated as the mean of valid days within each regimen. A minimum of one valid day that coincided with both FGM and activPAL data was required in each experimental regimen for inclusion in the analysis.

Postprandial glucose responses during waking hours were calculated as glucose iAUC for 2 h following each of the breakfast, lunch and dinner meals. Incremental area under the curve for each period was calculated by subtracting the last glucose concentration prior to each meal from the total AUC for the 2-h period.

#### Sitting, standing and stepping

Sitting, standing, stepping (light and moderate-vigorous physical activity [MVPA]) and sit-upright transitions were measured continuously for each activity regimen using an activPAL monitor. The activPAL is reliable and validated for measuring these outcomes (Edwardson et al. 2016; Ryan et al. 2006; Stansfield et al. 2015). The device was attached waterproof to the skin on the midline of the anterior aspect of the right upper thigh with 10 × 10 cm adhesive hypoallergic thin plastic film (Hypafix, BSN medical Limited, UK).

ActivPAL data was processed using Processing PAL v1.4.060919 (University of Leicester, UK; available at https://github.com/UOL-COLS/ProcessingPAL). A wear day was considered valid if wear time was ≥ 10 h, ≥ 500 steps were recorded, and < 95% of time spent in one activity (sitting, standing, or stepping) (Edwardson et al. 2017; Winkler et al. 2016). Variables generated for investigation included waking wear time, time spent sitting, standing, stepping, light stepping and MVPA stepping, total steps, light steps and MVPA steps. The number and time spent in sitting bouts of 0–30, 30–60, ≥ 30, and > 60-min, in addition to the number of sit-upright transitions were also generated. Each of these variables were calculated as the mean of valid days within each activity regimen.

Two criteria were used to evaluate participant compliance with each regimen protocol. In SIT, criteria were (1) standing time ≤ 1 h/day and (2) stepping time ≤ 1 h/day. In SITless the criteria were (1) standing time ≥ 3 h/day and (2) stepping time ≥ 2 h/day.

### Statistical analysis

Statistical analyses were performed using SPSS version 26.0 (SPSS Inc., Armonk, N.Y., USA). Data were tested for normality using Q-Q plots. Linear mixed models with a compound symmetry correlation structure were used to determine the main effect of experimental regimen (SIT vs. SITless) for the outcome variables, with experimental regimen included as a fixed factor and participants included as a random factor. The models analysing activPAL outcomes were adjusted for activPAL wear time. The models analysing FGM outcomes adjusted for BMI. Two-tailed statistical significance was set at *p* ≤ 0.05. Cohen’s *d* effect sizes were calculated, with 0.2, 0.5, and 0.8 indicating a small, medium or large effect, respectively (Cohen 1988). All data are presented as mean (95% confidence interval [CI]) unless stated otherwise.

## Results

Recruitment of participants and data collection took place between November 2020 and July 2021, see Supplementary Material S1 for the flow of participants. Following screening, 18 participants were enrolled, with four participants unable to fully complete the study protocol due to testing positive for COVID-19. Descriptive characteristics of the remaining 14 participants that were analysed are presented in Table 1.

**Table 1:** Participant characteristics

Characteristic	*N* = 14
Ethnic origin	
Bangladeshi	5 (36)
Indian	5 (36)
Pakistani	4 (28)
Religion	
Hinduism	4 (29)
Sikhism	3 (21)
Muslim	7 (50)
Employment status	
Employed	13 (93)
Retired	1 (7)
Female	7 (50)
Male	7 (50)
Age (years)	41 ± 3
Height (cm)	161.5 ± 2.1
Body mass (kg)	69.1 ± 2.4
Body mass index (kg^.^m^−2^)	26.5 ± 0.8
Waist circumference (cm)	92.3 ± 2.7
Fasting glucose (mmol.L^−1^)	5.3 ± 0.3

Data presented as mean ± standard error of the mean, or *N* (%)

### Flash glucose metrics

#### 24-h interstitial glucose

The 24-h FGM glucose metrics for the SIT and SITless regimens are presented in Table 2, where there were no significant differences in any of the variables between the two experimental regimens. There was a trend for a reduction in mean interstitial glucose in SITless (− 0.2 [95% CI − 0.5, 0.0] mmol.L^−1^) compared with SIT (*p* = 0.06), with a small effect size (*d* = 0.16). Supplementary Material S2 shows mean glucose concentrations across the 24 h day for each regimen.

**Table 2:** 24 h Flash glucose monitoring metrics for the SIT and SITless regimens (*N* = 14)

	SIT regimen	SITless regimen	*p-*value for main effect of regimen	Cohens’ *d* effect size
Mean interstitial glucose (mmol.L^−1^)	6.4 (5.5, 7.3)	6.2 (5.3, 7.1)	0.06	0.16
Glycaemic variability (%CV)	0.2 (0.2, 0.2)	0.2 (0.2, 0.2)	0.72	0.07
Time in range (3.9–10.0 mmol.L^−1^) (%)	91.7 (84.9, 98.6)	94.1 (87.3, 101.0)	0.30	0.20
Time above range (> 10.0 mmol.L^−1^) (hyperglycaemia) (%)	6.9 (− 1.7, 15.4)	4.6 (− 3.9, 13.2)	0.10	0.15
Time below range (< 3.0 mmol.L^−1^) (hypoglycaemia) (%)	2.3 (− 0.3, 4.9)	2.0 (− 0.6, 4.6)	0.88	0.06
Interstitial glucose iAUC (mmol.L^−1^.24 h^−1^)	140.0 (119.4, 160.5)	137.3 (116.8, 157.8)	0.40	0.07

Data presented as mean (95% confidence interval); iAUC, net incremental area under the curve

#### Waking hour interstitial glucose

The FGM metrics for normalised 16-h waking data in SIT and SITless are shown in Table 3. Interstitial glucose iAUC was significantly lower by − 9.2 mmol.L^−1^·16 h^−1^ (95% CI − 18.1, − 0.3) .L^−1^·16 h^−1^ in SITless compared with SIT (*p* = *0.04)*. There was a trend for a reduction in mean interstitial glucose (− 0.6 mmol.L^−1^; 95% CI − 1.3, 0.1, *p* = 0.07) and lower time above range (− 2.7 mmol.L^−1^; 95% CI − 5.5, 0.2, *p* = 0.06) in SITless compared with SIT. Supplementary Material S3 shows mean glucose concentrations across the normalised 16-h waking day for each regimen.

**Table 3:** Flash glucose monitoring metrics for waking hours (16 h) in the SIT and SITless regimens (*N* = 14)

	SIT regimen	SITless regimen	*p*-value for the main effect of regimen	Cohens’ *d* effect size
Mean interstitial glucose (mmol.L^−1^)	7.2 (6.0, 8.3)	6.6 (5.4, 7.7)	0.07	0.31
Glycaemic variability (%CV)	0.2 (0.2, 0.2)	0.2 (0.2, 0.2)	0.61	0.11
Time in range (3.9–10.0 mmol.L^−1^) (%)	97.6 (88.0, 107.2)	95.1 (85.5, 104.7)	0.56	0.15
Time above range (> 10.0 mmol.L^−1^) (hyperglycaemia) (%)	9.4 (− 1.5, 20.2)	6.7 (− 4.1, 17.5)	0.06	0.14
Time below range (< 3.0 mmol.L^−1^) (hypoglycaemia) (%)	0.4 (− 1.0, 1.9)	2.2 (0.7, 3.6)	0.10	0.66
Interstitial glucose iAUC (mmol.L^−1^·16 h^−1^)	98.5 (82.7, 114.4)	89.4 (73.5, 105.2)	**0.04**	0.33

Data presented as mean (95% confidence interval); iAUC, net incremental area under the curve. Statistically significant (*p* ≤ 0.05) differences highlighted in bold

#### Postprandial glucose responses

**Fig. 2:**
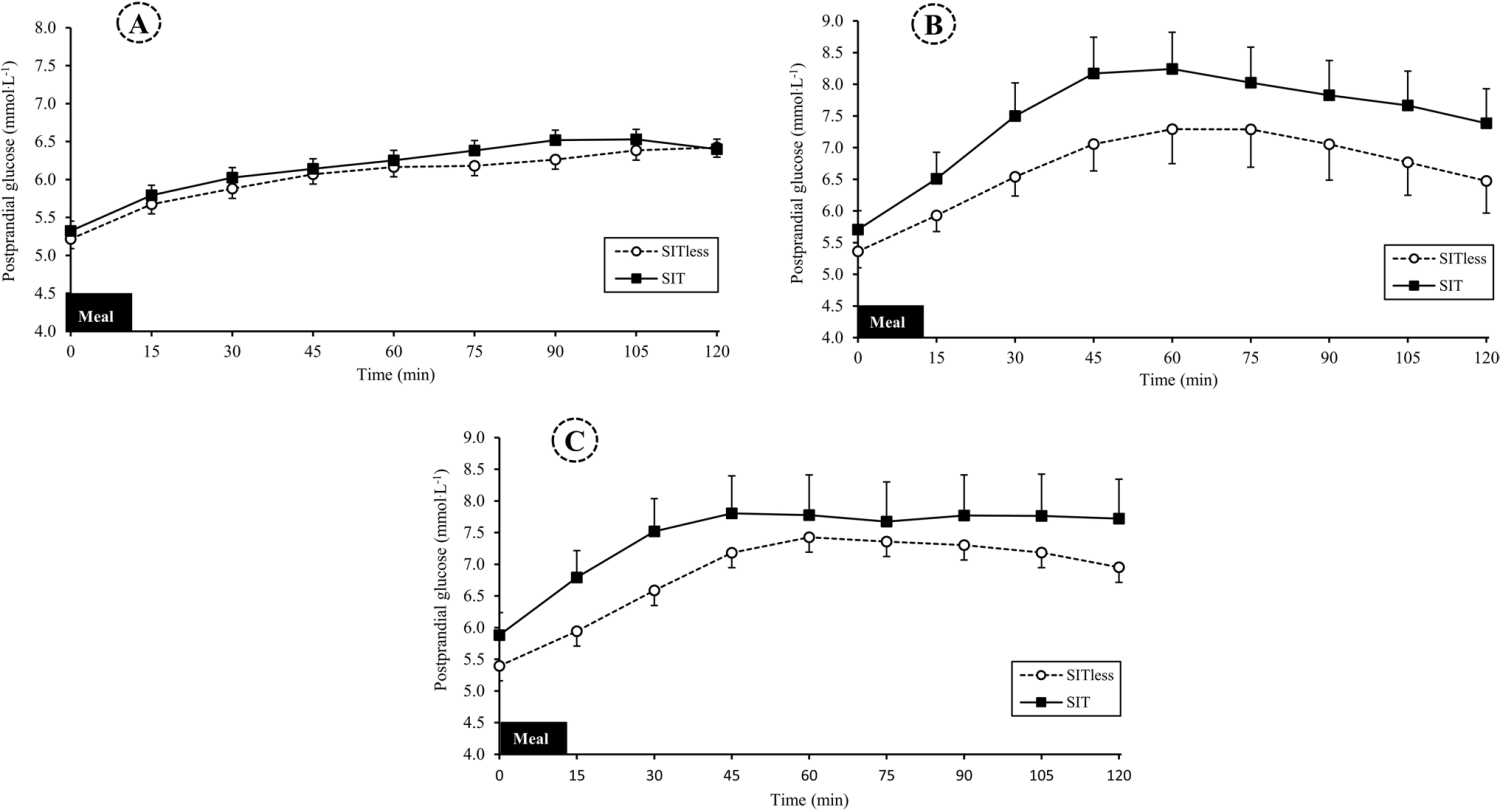
Postprandial interstitial glucose concentrations for (**A**) breakfast period, 0.06 (95% CI: − 0.67, 0.79) and − 0.06 (95% CI − 0.79, 0.67) mmol.L^−1^·2 h^−1^ in SIT and SITless, respectively, (B) lunch period, 0.99 (95% CI 0.20, 1.78) and − 0.99 (95% CI − 1.78, − 0.20) mmol.L^−1^·2 h^−1^ in SIT and SITless, respectively, (C) dinner period, 0.21 (95% CI − 0.92, 1.35) and − 0.21 (95% CI − 1.35, 0.92) mmol.L^−1^·2 h^−1^ in SIT and SITless, respectively. Data are mean and 95% confidence interval

Postprandial glucose responses following the breakfast, lunch, and dinner meals are presented in Fig. 2. Compared to SIT, lunch postprandial glucose iAUC was significantly lower by − 1.0 mmol.L^−1^·2 h^−1^ (95% CI − 1.8, 0.2) in SITless (*p* = 0.02; *d* = 0.48). (Supplementary Material S4). There were no differences in 2 h postprandial glucose iAUC response following breakfast (*p* = 0.86; *d* = 0.06) or dinner meals (*p* = 0.69; *d* = 0.09).

### Sitting, standing and stepping

Sitting time was lower by − 3.6 h/day (95% CI − 4.9, − 2.3) in SITless compared with SIT (Table 4). Compared to SIT, time in sitting bouts lasting ≥ 30 min was lower by − 3.0 h/day (95% CI − 4.3, − 1.6) and the number of sedentary bouts lasting > 30-min was lower by − 2.0 per day (95% CI − 3.2, − 0.9) in SITless (Table 4). Standing time was higher by 1.9 h/day (95% CI 0.6, 3.2) and total stepping time was 1.6 h/day (95% CI 1.2, 2.1) higher in SITless. Light and MVPA stepping time were higher by 0.5 h/day (95% CI 0.4, 0.6) and 1.2 h/day (95% CI 0.8, 1.6) in the SITless regimen, respectively. Participants accumulated an additional 8698 steps/day (95% CI 5841, 11,555) in SITless compared with SIT.

**Table 4:** Sitting, standing and stepping outcomes for the SIT and SITless regimens (*N* = 14)

	SIT regimen	SITless regimen	*p*-value for the main effect of regimen	Cohens’ *d* effect size
Sitting (h/day)	10.6 (9.5, 11.7)	7.0 (5.9, 8.1)	**< 0.01**	1.65
Time in sedentary bouts lasting 0–30 min (h/day)	3.4 (2.8, 4.1)	3.4 (2.7, 4.0)	0.84	0.03
Time in sedentary bouts lasting 30–60 min (h/day)	2.2 (1.6, 2.7)	1.6 (1.0, 2.2)	0.11	0.51
Time in sedentary bouts lasting ≥ 60 min (h/day)	5.1 (3.6, 6.5)	2.7 (1.3, 4.1)	**< 0.01**	0.93
Time in sedentary bouts lasting ≥ 30 min (h/day)	7.3 (5.7, 8.8)	4.3 (2.7, 5.8)	**< 0.01**	1.08
Number of sedentary bouts lasting 0–30 min per day	38.5 (29.5, 47.5)	42.7 (33.7, 51.7)	0.27	0.31
Number of sedentary bouts lasting 30–60 min per day	3.0 (2.2, 3.8)	2.3 (1.5, 3.1)	0.15	0.48
Number of sedentary bouts lasting > 60 min per day	2.8 (2.1, 3.6)	1.5 (0.8, 2.3)	**0.01**	0.99
Number of sedentary bouts lasting > 30 min per day	5.8 (4.7, 7.0)	3.8 (2.7, 4.9)	**< 0.01**	0.97
Total number of sedentary bouts per day	44.2 (35.7, 52.6)	46.5 (38.1, 55.0)	0.50	0.22
Number of sit-upright transitions per day	43.9 (35.5, 52.3)	46.3 (37.9, 54.7)	0.49	0.22
Standing time (h/day)	3.8 (2.8, 4.8)	5.7 (4.8, 6.7)	**0.01**	1.08
Stepping time (h/day)	1.2 (0.8, 1.5)	2.8 (2.4, 3.1)	**< 0.01**	2.39
Light stepping time (h/day)	0.6 (0.3, 0.8)	1.0 (0.8, 1.2)	**< 0.01**	1.32
Moderate-to-vigorous stepping time (h/day)	0.6 (0.3, 0.9)	1.8 (1.5, 2.1)	**< 0.01**	2.25
Number of light steps per day	1226 (754, 1698)	2398 (1926, 2870)	**< 0.01**	1.47
Number of moderate-to-vigorous steps per day	3709 (1657, 5761)	11,314 (9252, 13,375)	**< 0.01**	2.15
Total number of steps per day	4960 (2828, 7092)	13,658 (11,516, 15,799)	**< 0.01**	2.33

Data presented as mean (95% confidence interval); Statistically significant (*p* ≤ 0.05) differences highlighted in bold

In SITless, 71% of participants (*N* = 10) complied fully with the regimen criteria across all four days, two participants complied fully for three days, and two participants complied fully for one day (Supplementary Material S5). Only 7% participants (*N* = 1) complied fully with the SIT regimen criteria across all four days, one participant complied fully for three days, and two participants complied fully for two days (Supplementary Material S6).

## Discussion

This is the first study to evaluate continuously monitored glucose concentrations in response to substituting sitting under free-living conditions in South Asian adults with overweight and obesity. The main finding was that glycaemia during waking hours was reduced during a 4-day period of substituting sitting with standing and walking versus an equivalent period of predominantly sitting. These attenuated glucose responses are in contrast to previous free-living studies in Caucasian individuals with overweight and obesity, which did not observe improvements in continuous glucose or oral glucose tolerance with four days of substituting sitting with standing and stepping (Bailey et al. 2022; Duvivier et al. 2013). There were greater reductions in sitting (3.6 h/day) and prolonged sitting (3.0 h/day) in the substituting sitting with standing and walking condition versus the sitting condition in the present study than those seen in Bailey et al. (2022) (58 min/day and 99 min/day, respectively), which could explain the disparity in findings. Furthermore, differences in participant characteristics (e.g. fitness, physical activity levels and baseline insulin sensitivity) between studies could be other explanatory factors (McCarthy et al. 2017). Using a similar design, improvements in insulin sensitivity have been observed in previous research (Duvivier et al. 2017a). It is unknown whether insulin sensitivity was affected in the current study, as biomarkers of cardiometabolic health other than glucose were not assessed. Collectively, the absence of additional biomarker data restricts a more comprehensive assessment of metabolic health in this population. Future research should include a broader panel of metabolic biomarkers, including insulin, to advance understanding of cardiometabolic responses and to support the development of more targeted and effective interventions.

Substituting sitting over a longer period could have had more widespread cardiometabolic effects than those reported here. Indeed, Smith et al (2021) aimed to interrupt sitting with 3 min of LPA or MVPA every 30 min and reported reduced fasting glucose and glucose variability in obese individuals under free-living conditions over three weeks (Smith et al. 2021). These findings may be surprising because this study was unsuccessful in increasing the number of sit-upright transitions or reducing daily sitting and, although a 10.4 min/day increase in walking time was shown (Smith et al. 2021), our study reported a much larger increase in walking of 1.6 h/day. As such, sustaining changes in sitting, standing and stepping over a longer duration may be particularly important for inducing improvements in other glucose metrics. Future research should evaluate the effects of substituting sitting over the longer term in South Asian individuals.

Reducing the volume of sitting and replacing this with 1.9 and 1.6 h of standing and stepping, respectively, was sufficient for attenuating waking hour and post-lunch glucose concentrations. The reductions in glucose may be a consequence of increased muscle contractile activity leading to augmented GLUT4 translocation and skeletal muscle glucose uptake (Homer et al. 2019). Yet, the behavioural changes did not lead to improvements in 24 h glucose metrics, other than a trend for lower mean interstitial glucose. The effect size used to inform the sample size was based on single-day laboratory studies with overweight and obese adults, where the effect is most likely larger than in free-living studies due to the controlled supervised conditions and assessment of the immediate postprandial response to interrupting sitting. An effect this large may not be achieved over longer unsupervised free-living monitoring periods. However, free-living data in adults with overweight and obesity were not available to inform our sample size calculation. Furthermore, although participants were able to substitute their sitting, they did not fully comply with the protocols. In particular, participants found it difficult to keep their standing and stepping to < 1 h/day each to comply with the SIT regimen. On average, participants needed to substitute a further 1.5 h per day of sitting to be compliant, which may account for limited effects on 24 h glucose metrics. That said, the difference in sitting between the regimens was sufficient for attenuating daily and postprandial glucose responses. As postprandial glucose is a strong independent predictor of CVD (Wang et al. 2024), our findings can inform intervention strategies for promoting cardiometabolic health in South Asian individuals living with overweight/obesity. There is a plethora of evidence demonstrating that reductions in postprandial glucose during an oral glucose tolerance test are associated with significantly lower risk of CVD and Type 2 diabetes (Ceriello 2004), meaning that the present findings have clinical relevance. The longer-term benefits of the glucose attenuation observed here in response to free-living mixed meals require future investigation to better inform the relevance of this intervention strategy for managing glycaemia.

During the SITless regimen, sitting was lower by 3.6 h per day with a 3.0 h per day reduction in prolonged sitting. This indicates that participants appeared to adapt their sitting bout durations in order to comply with the guideline volumes for sitting, standing and stepping. These are similar to previous findings in overweight and obese participants, whereby daily and prolonged sitting were 58 and 99 min per day lower, respectively, than an imposed sitting regimen under free-living conditions (Bailey et al. 2022). Smith et al (2021) reported no changes in daily sitting or sitting bouts in sedentary individuals over three weeks compared to habitual baseline monitoring. The current study demonstrates the ability to substitute sitting over the short term in South Asian individuals with overweight and obesity. Yet, it was not possible to manipulate interruptions in sitting (sit-upright transitions) under free-living conditions in our study and previous free-living studies (Bailey et al. 2022; Duvivier et al. 2017b; Smith et al. 2021), which is likely due to participants not being under close supervision from the research team. An inability to manipulate interruptions in sitting under free-living conditions may limit the potential effectiveness of sedentary behaviour interventions on glucose concentrations and requires consideration in future work. Chronic intervention studies are also needed, informed by the present study, to explore the potential to achieve the sitting-related behavioural changes over the longer term.

This study was conducted under free-living conditions. Thus, given the applied nature, the findings are more ecologically valid than controlled laboratory settings. Furthermore, an in-depth understanding of the effects of substituting sitting on a range of 24-h and waking hour glucose metrics was possible through the use of glucose monitoring via FGM. An additional strength was the continuous monitoring of sitting, standing and stepping throughout the regimens to evaluate manipulations in these variables. A potential limitation was the absence of baseline habitual measures of sitting, standing and stepping for direct comparison of the outcomes to usual behaviour. Direct measures of body fat were also not obtained due to COVID-19 restrictions, limiting the ability to assess more precise, health-related indicators of obesity. Further, compliance to the protocols was not achieved fully, so future research should implement additional strategies to maximise this. The study also took place during the COVID-19 pandemic, which could have affected the extent to which participants manipulated their sitting and physical activity.

## Conclusions

In conclusion, these findings demonstrate that substituting sitting with standing and walking reduces glucose concentrations during waking hours under free-living conditions in South Asian individuals with overweight and obesity. The results highlight the potential of substituting sitting as an intervention strategy for improving cardiometabolic health in this population group. Further research is needed to explore the longer-term cardiometabolic effects of this behavioural approach in South Asian individuals.

## Supplementary Information

Below is the link to the electronic supplementary material.

Supplementary file1 (DOCX 602 KB)

## Data Availability

The datasets generated during the current study are available from the corresponding author (daniel.bailey@brunel.ac.uk).

## Abbreviations

BMI: Body mass index
CGM: Continuous glucose monitoring
CI: Confidence interval
CVD: Cardiovascular disease
FGM: Flash glucose monitor
GI: Glycaemic index
i-AUC: Incremental area under the curve
LPA: Light-intensity physical activity
MVPA: Moderate-vigorous physical activity
OGTT: Oral glucose tolerance test
