# Supplementary material for: Substituting sitting with standing and walking in free-living conditions improves daily glucose concentrations in South Asian adults living with overweight/obesity
Source: Eur J Appl Physiol. 2025 Aug 5;126(1):579–89. doi: 10.1007/s00421-025-05919-7 (PMC12881178; doi:10.1007/s00421-025-05919-7)
Supplement: Supplementary file 1 — Supplementary file1 (DOCX 602 KB) [file 421_2025_5919_MOESM1_ESM.docx]

**Supplementary Material S1.** Participant flow throughout the study.


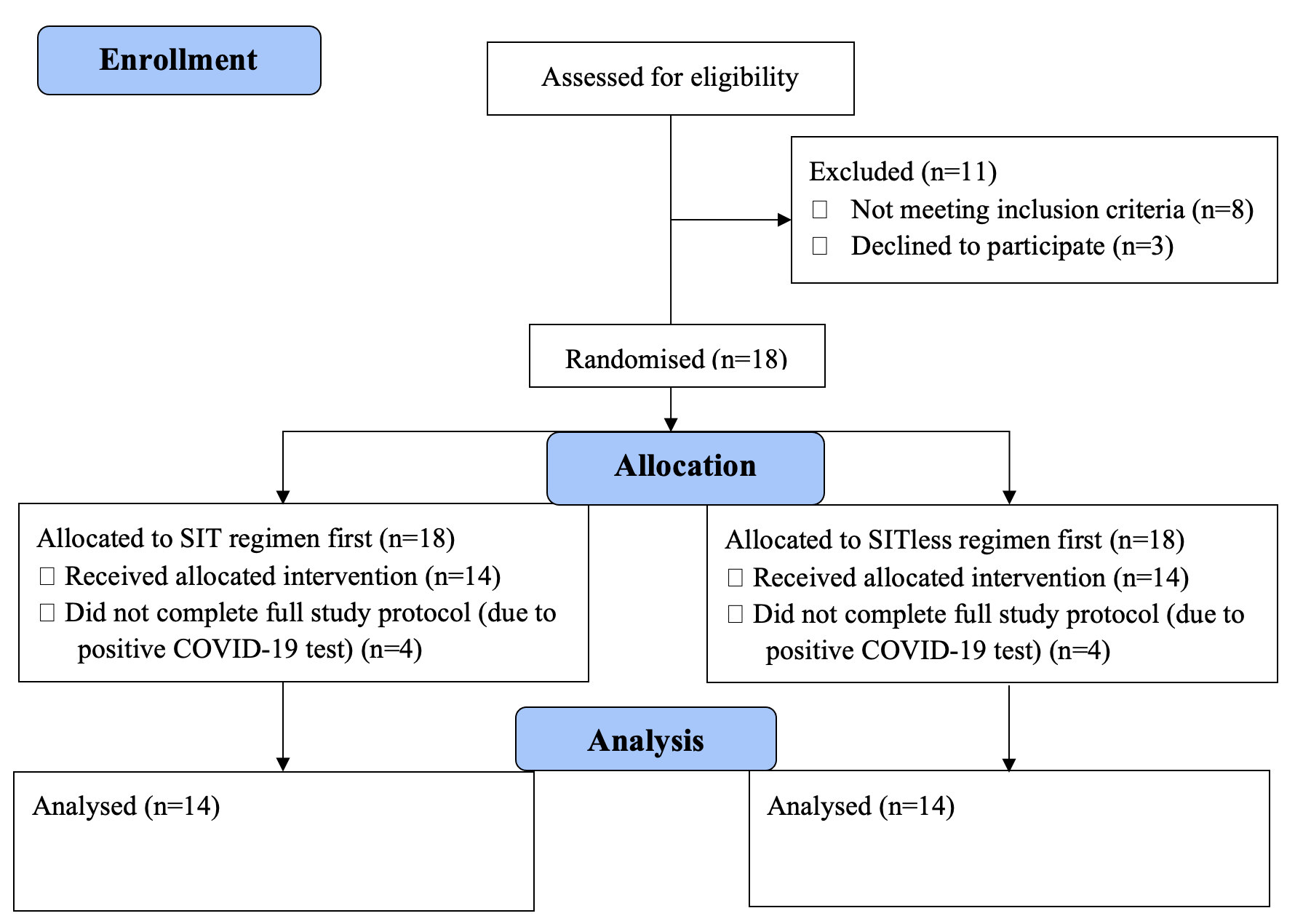


**Supplementary Material S2.** Mean 24-h interstitial glucose concentration for the SIT and SITless regimens.


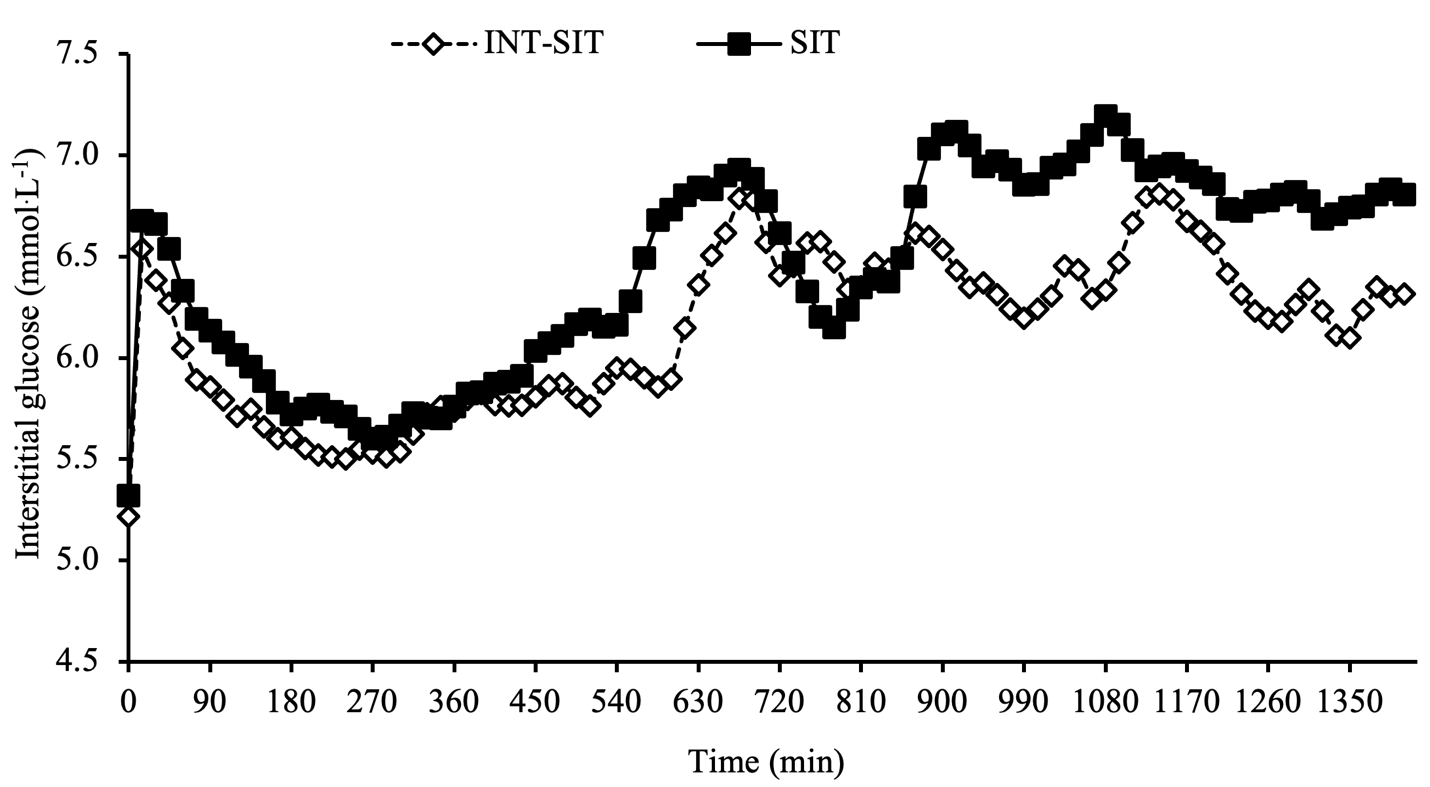


**Supplementary Material S3.** Mean waking hour (normalised to a 16-h day) interstitial glucose concentration for the SIT and SITless regimens.

**
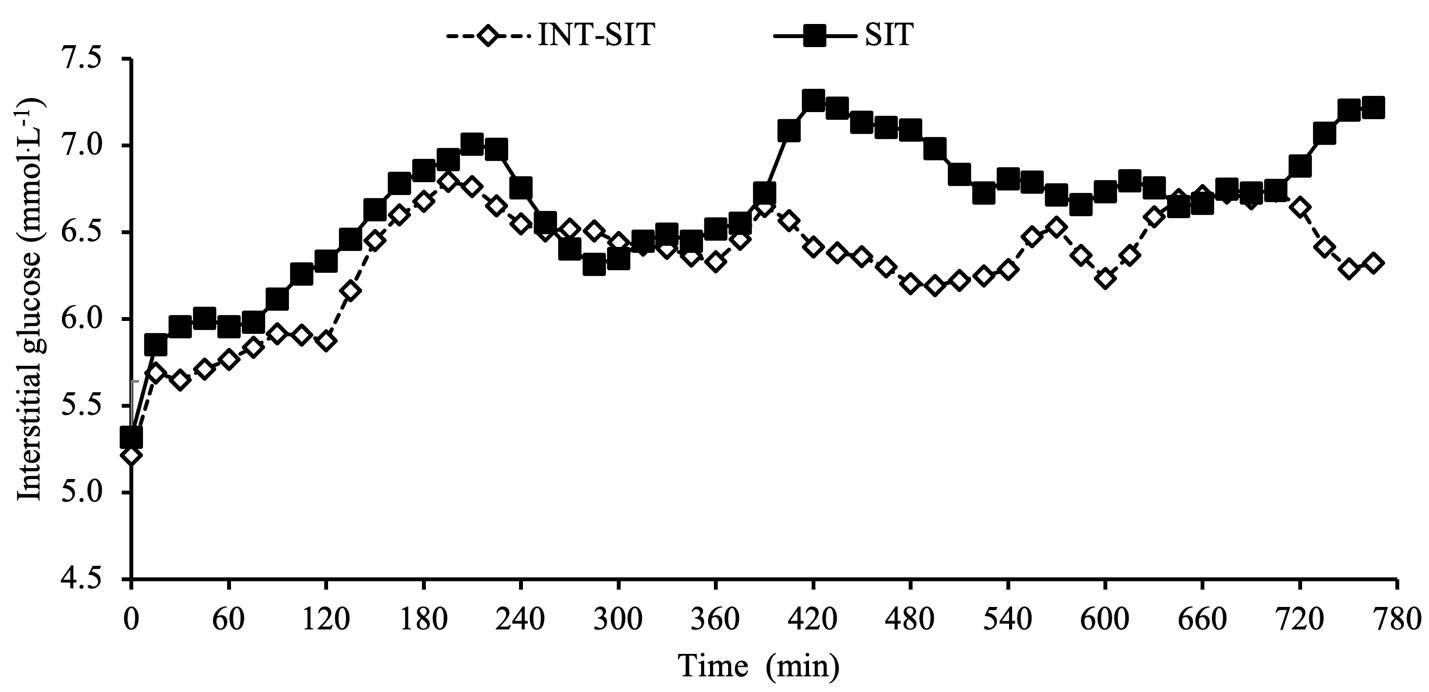
**

**Supplementary Material S4.** Post-prandial interstitial glucose concentration net-incremental area under the curve responses in the SIT and SITless regimens. Data are mean and 95% confidence interval. Significant main effect of regimen for the lunch postprandial glucose response (*p*=0.02; *d=*0.48).


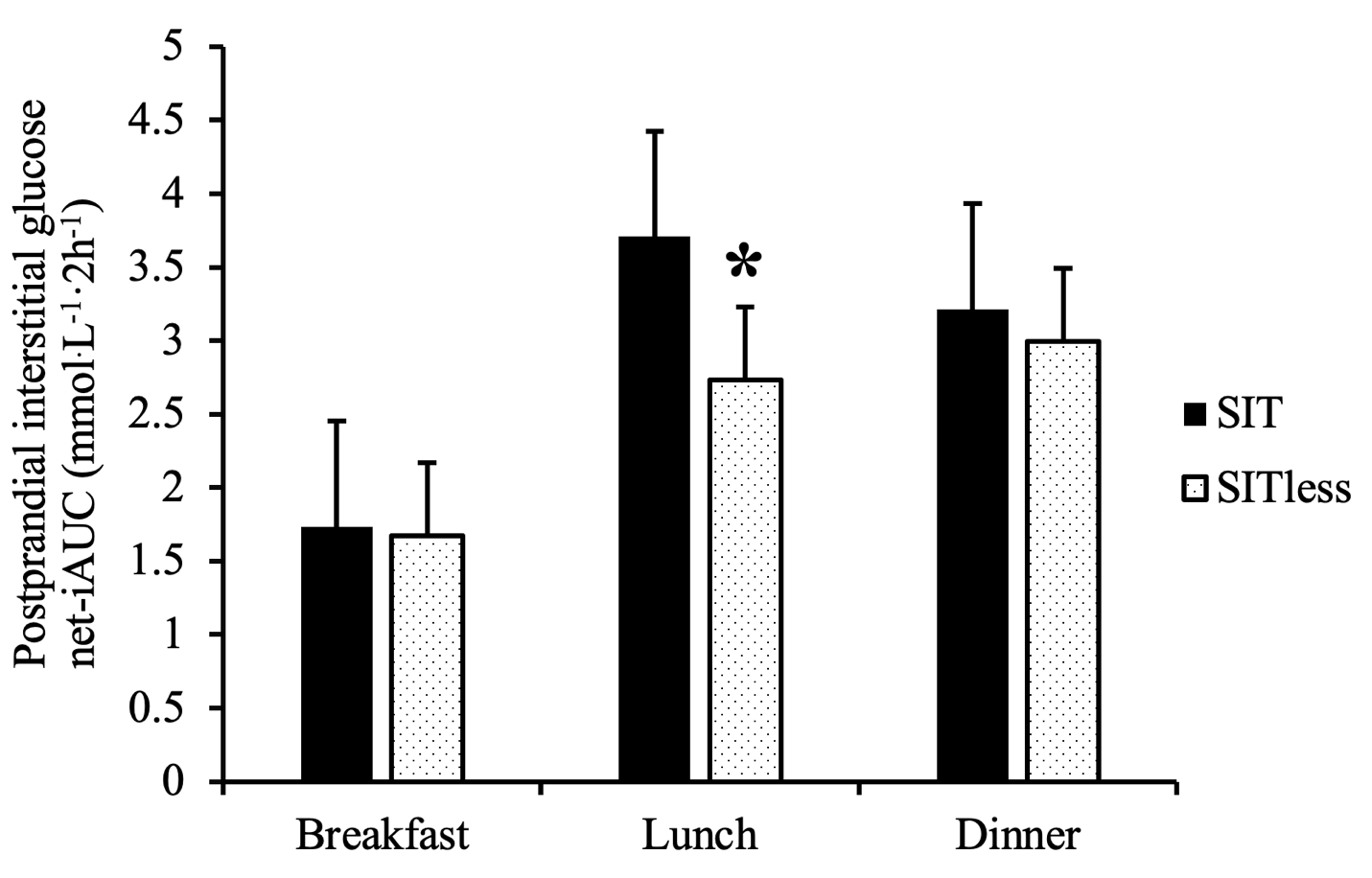


**Supplementary Material S5.** Compliance with SITless regimen criteria.

| **Participant ID** | **Day** | **Criteria 1 (total standing time ≥3 h/day)** | **Criteria 2 (total stepping time ≥2 h/day)** | **Total number of compliance criteria (score)** |
| --- | --- | --- | --- | --- |
| 01 | 1 | Yes | Yes | 2 |
|  | 2 | Yes | Yes | 2 |
|  | 3 | Yes | Yes | 2 |
|  | 4 | Yes | Yes | 2 |
| 02 | 1 | Yes | Yes | 2 |
|  | 2 | Yes | Yes | 2 |
|  | 3 | Yes | Yes | 2 |
|  | 4 | Yes | Yes | 2 |
| 03 | 1 | Yes | Yes | 2 |
|  | 2 | Yes | Yes | 2 |
|  | 3 | Yes | Yes | 2 |
|  | 4 | Yes | Yes | 2 |
| 04 | 1 | No | Yes | 1 |
|  | 2 | Yes | Yes | 2 |
|  | 3 | Yes | No | 1 |
|  | 4 | Yes | No | 1 |
| 05 | 1 | Yes | Yes | 2 |
|  | 2 | Yes | Yes | 2 |
|  | 3 | Yes | Yes | 2 |
|  | 4 | Yes | yes | 2 |
| 06 | 1 | Yes | Yes | 2 |
|  | 2 | Yes | Yes | 2 |
|  | 3 | Yes | Yes | 2 |
|  | 4 | Yes | Yes | 2 |
| 07 | 1 | Yes | Yes | 2 |
|  | 2 | Yes | Yes | 2 |
|  | 3 | Yes | Yes | 2 |
|  | 4 | Yes | Yes | 2 |
| 08 | 1 | Yes | Yes | 2 |
|  | 2 | Yes | Yes | 2 |
|  | 3 | Yes | Yes | 2 |
|  | 4 | Yes | Yes | 2 |
| 09 | 1 | Yes | Yes | 2 |
|  | 2 | Yes | Yes | 2 |
|  | 3 | Yes | Yes | 2 |
|  | 4 | Yes | Yes | 2 |
| 10 | 1 | Yes | Yes | 2 |
|  | 2 | Yes | Yes | 2 |
|  | 3 | Yes | Yes | 2 |
|  | 4 | No | Yes | 1 |
| 12 | 1 | Yes | Yes | 2 |
|  | 2 | Yes | Yes | 2 |
|  | 3 | Yes | Yes | 2 |
|  | 4 | Yes | Yes | 2 |
| 13 | 1 | Yes | Yes | 2 |
|  | 2 | Yes | Yes | 2 |
|  | 3 | No | No | 0 |
|  | 4 | Yes | Yes | 2 |
| 14 | 1 | Yes | Yes | 2 |
|  | 2 | Yes | Yes | 2 |
|  | 3 | Yes | yes | 2 |
|  | 4 | Yes | Yes | 2 |
| 16 | 1 | Yes | Yes | 2 |
|  | 2 | No | Yes | 1 |
|  | 3 | No | Yes | 1 |
|  | 4 | No | Yes | 1 |

“Yes” was scored 1 and “No” was scored 0.

Yellow highlight indicates where participants fully complied with the criteria. Based on the compliance criteria, only one participant (ID 13) complied fully (a total of two criteria) for all four days, one participant (ID10) complied fully for three days, and two participants (ID 09 and 16) complied fully for two days. Overall, 7% participants complied fully with the SIT regimen protocol.

**Supplementary Material S6.** Compliance with SIT regimen criteria.

| **Participant ID** | **Day** | **Criteria 1 (total standing time ≤1 h/day)** | **Criteria 2 (total stepping time ≤1 h/day)** | **Total number of compliance criteria (score)** |
| --- | --- | --- | --- | --- |
| 01 | 1 | No | No | 0 |
|  | 2 | No | No | 0 |
|  | 3 | No | Yes | 1 |
|  | 4 | No | No | 0 |
| 02 | 1 | No | No | 0 |
|  | 2 | No | No | 0 |
|  | 3 | No | No | 0 |
|  | 4 | No | No | 0 |
| 03 | 1 | No | No | 0 |
|  | 2 | No | No | 0 |
|  | 3 | No | No | 0 |
|  | 4 | No | Yes | 1 |
| 04 | 1 | No | Yes | 1 |
|  | 2 | No | Yes | 1 |
|  | 3 | No | Yes | 1 |
|  | 4 | No | Yes | 1 |
| 05 | 1 | No | Yes | 1 |
|  | 2 | No | Yes | 1 |
|  | 3 | No | Yes | 1 |
|  | 4 | No | yes | 1 |
| 06 | 1 | No | No | 0 |
|  | 2 | No | No | 0 |
|  | 3 | No | No | 0 |
|  | 4 | No | No | 0 |
| 07 | 1 | No | Yes | 1 |
|  | 2 | No | Yes | 1 |
|  | 3 | No | Yes | 1 |
|  | 4 | No | No | 1 |
| 08 | 1 | No | No | 1 |
|  | 2 | No | No | 1 |
|  | 3 | No | No | 1 |
|  | 4 | No | No | 1 |
| 09 | 1 | No | Yes | 1 |
|  | 2 | Yes | Yes | 2 |
|  | 3 | Yes | Yes | 2 |
|  | 4 | No | Yes | 1 |
| 10 | 1 | Yes | Yes | 2 |
|  | 2 | No | Yes | 1 |
|  | 3 | Yes | Yes | 2 |
|  | 4 | Yes | Yes | 2 |
| 12 | 1 | No | Yes | 1 |
|  | 2 | No | Yes | 1 |
|  | 3 | No | Yes | 1 |
|  | 4 | No | Yes | 1 |
| 13 | 1 | Yes | Yes | 2 |
|  | 2 | Yes | Yes | 2 |
|  | 3 | Yes | Yes | 2 |
|  | 4 | Yes | Yes | 2 |
| 14 | 1 | No | Yes | 1 |
|  | 2 | No | No | 0 |
|  | 3 | No | No | 0 |
|  | 4 | No | Yes | 1 |
| 16 | 1 | No | No | 0 |
|  | 2 | No | Yes | 1 |
|  | 3 | Yes | Yes | 2 |
|  | 4 | Yes | Yes | 2 |

“Yes” was scored 1 and “No” was scored 0.

Yellow highlight indicates where participants fully complied with the criteria. Based on the compliance criteria, ten participants (ID 01, 02, 03, 05, 06, 07, 08, 09, 12, and 14) complied fully with the criteria for four days, two participants (ID 10 and 13) complied fully for three days, and two participants (ID 04, and 16) complied fully for one day in SITless. Overall, 71% participants complied fully with the SITless regimen protocol.
